# Recent Progress in Hyaluronic-Acid-Based Hydrogels for Bone Tissue Engineering

**DOI:** 10.3390/gels9070588

**Published:** 2023-07-21

**Authors:** Hee Sook Hwang, Chung-Sung Lee

**Affiliations:** 1 Department of Pharmaceutical Engineering, Dankook University, Cheonan 31116, Republic of Korea; 2 Department of Pharmaceutical Engineering, Soonchunhyang University, Asan 31538, Republic of Korea

**Keywords:** hyaluronic acid, hydrogel, scaffold, bone tissue engineering, bone regeneration

## Abstract

Hydrogel-based bone tissue engineering is a potential strategy for treating bone abnormalities and fractures. Hyaluronic acid (HA) is a natural polymer that is widely distributed in the human body and plays a significant role in numerous physiological processes such as cell migration, tissue hydration, and wound healing. Hydrogels based on HA and its derivatives have gained popularity as potential treatments for bone-related diseases. HA-based hydrogels have been extensively studied for their ability to mimic the natural extracellular matrix of bone tissue and provide a suitable microenvironment for cell support and tissue regeneration. The physical and chemical properties of HA can be modified to improve its mechanical strength, biocompatibility, and osteogenic potential. Moreover, HA-based hydrogels combined with other biomaterials in the presence or absence of bioactive agents have been investigated as a means of improving the mechanical properties and bioactivity of the hydrogel scaffold. Therefore, HA-based hydrogels have shown great promise in bone tissue engineering due to their biocompatibility, osteogenic activity, and ability to mimic the natural extracellular matrix of bone tissue. Overall, this review provides a comprehensive overview of the current state of the art in HA-based hydrogels for bone tissue engineering, highlighting the key advances, challenges, and future directions in this rapidly evolving field.

## 1. Introduction

Bone tissue engineering is an emerging field that aims to regenerate damaged or diseased bone tissue using biomaterials, cells, and growth factors to restore normal skeletal function [1,2]. The field has grown rapidly in recent years, driven by the increasing prevalence of bone disorders and injuries such as osteoporosis, bone fractures, and bone defects caused by trauma or disease [3]. The current treatments for these conditions, such as bone grafting and implantation of metal or ceramic implants, have limitations and drawbacks, including limited availability of donor tissue, risk of infection, and poor integration with surrounding tissues.

Hydrogels have emerged as promising biomaterials for bone tissue engineering applications due to their ability to mimic the extracellular matrix (ECM) of bone tissue and their tunable physical and chemical properties [4]. Hydrogels are three-dimensional networks of hydrophilic polymer chains that can absorb large amounts of water while maintaining their structural integrity [5]. Hydrogels can be designed and formulated to provide a suitable microenvironment for bone cells and promote bone tissue regeneration.

**Figure 1:**
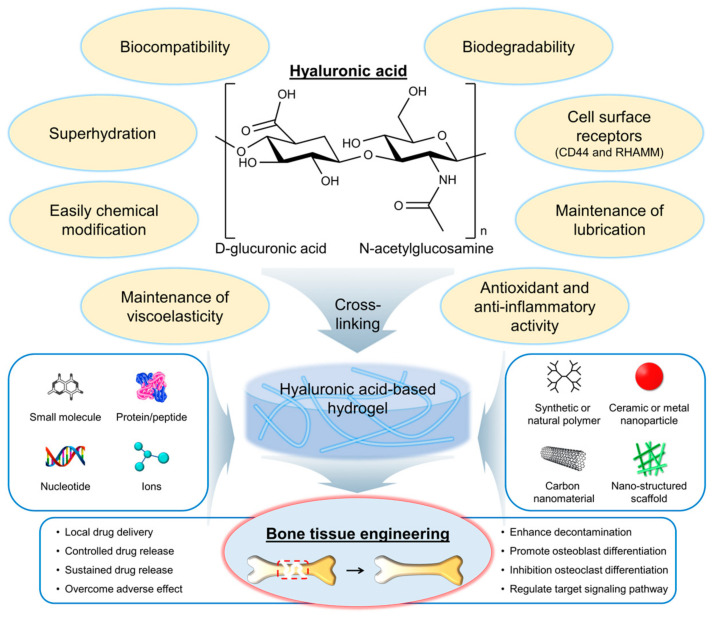
Chemical structure and properties of hyaluronic acid (HA), as well as its application in the development of hydrogels for bone tissue engineering. Red dashed box indicates the bone defect and injury area. RHAMM, a receptor for hyaluronan-mediated motility.

Hyaluronic acid (HA) is a naturally occurring polysaccharide that has emerged as a promising biomaterial for bone regeneration due to its biocompatibility, biodegradability, and ability to interact with the cells and growth factors involved in bone formation [6,7]. HA is a linear molecule consisting of repeating disaccharide units of glucuronic acid and *N*-acetylglucosamine (Figure 1) [8]. The molecular weight of HA can vary widely, from a few hundred to millions of Daltons. One of the key features of HA that make it an attractive biomaterial for bone regeneration is its ability to interact with the cells and growth factors involved in bone formation. HA can bind to cell surface receptors, such as CD44 and the receptor for hyaluronan-mediated motility (RHAMM), that are expressed on osteoblasts and osteoclasts, as well as on mesenchymal stem cells (MSCs), and that can differentiate into bone-forming cells [9]. HA can also interact with growth factors, such as bone morphogenetic proteins (BMPs) and transforming growth factor-beta (TGF-β), which play important roles in bone formation and regeneration [10].

HA has several properties and characteristics that make it a promising biomaterial for bone tissue engineering applications (Figure 1) [11,12]. First, HA is biocompatible, meaning it does not have adverse effects on living tissues and cells. The biocompatibility of HA is due to its natural occurrence in the body and its nonimmunogenic nature [13]. This means that it is unlikely to cause an immune response in the body, reducing the risk of rejection. Second, HA is biodegradable, meaning it can be broken down and metabolized by the body over time. The biodegradability of HA is due to the presence of specific enzymes in the body, such as hyaluronidases, that can cleave the glycosidic bonds between the disaccharide units of HA [14]. The biodegradation of HA can be controlled by modifying the molecular weight and degree of cross-linking of the polymer chains. Third, HA can interact with the cells and growth factors involved in bone formation. As mentioned earlier, HA can bind to cell surface receptors, such as CD44 and RHAMM, which are expressed on osteoblasts and osteoclasts, as well as on MSCs, that can differentiate into bone-forming cells [9]. The interaction of HA with cells and growth factors can be modulated by modifying the physical and chemical properties of the HA. Fourth, HA is a highly hydrated molecule, allowing it to absorb large amounts of water and create a hydrophilic environment that is favorable for cell attachment and proliferation [15]. This makes HA-based hydrogels particularly well suited for tissue engineering applications because they can provide a suitable microenvironment for cell growth and differentiation. Finally, HA can be easily modified to introduce functional groups that can be used for cross-linking and the incorporation of bioactive molecules [16]. This allows for the creation of HA-based hydrogels with tailored physical and chemical properties such as mechanical strength, degradation rate, and bioactivity. Furthermore, HA is also known as a lubricant or support for other lubricants, such as lipids, in synovial joints [17]. Antioxidant activity and anti-inflammatory activity of HA have also been reported [18,19,20,21,22].

HA-based hydrogels have several advantages over other biomaterials for bone tissue engineering applications. The hydrophilic nature of HA-based hydrogels promotes the adsorption of BMPs and other growth factors that are involved in bone formation and regeneration [23]. The presence of these growth factors can enhance the osteogenic differentiation of MSCs and promote the formation of new bone tissue. In addition, the mechanical properties of HA-based hydrogels can be easily tuned by adjusting the degree of cross-linking and the molecular weight of the HA polymer chains [24]. This allows for the creation of hydrogels with a wide range of mechanical properties that are suitable for different applications. Hydrogels with higher mechanical strength may be used for load-bearing applications, whereas hydrogels with lower mechanical strength may be used for applications where flexibility and conformability are important. Furthermore, HA-based hydrogels can be easily modified to incorporate bioactive molecules, such as growth factors and ECM components, that can further enhance the osteogenic differentiation of MSCs and promote the formation of new bone tissue [25]. The incorporation of these bioactive molecules can also improve the integration of the hydrogel with surrounding tissues. Finally, HA-based hydrogels are biocompatible and biodegradable, meaning they are unlikely to cause adverse effects in the body and can be broken down and metabolized over time [26]. This reduces the risk of inflammation and other complications associated with the use of synthetic biomaterials.

The objective of this review is to provide an overview of recent progress in the development of HA-based hydrogels for bone tissue engineering applications. We review recent studies on the design and formulation of HA-based hydrogels for bone tissue engineering, including the incorporation of bioactive molecules and the modulation of physical and chemical properties. In addition, we discuss the challenges and future directions of HA-based hydrogels for bone tissue engineering, including the optimization of mechanical properties, the improvement of cell adhesion and proliferation, and the translation of these technologies into clinical practice.

## 2. Design and Formulation of Hyaluronic-Acid-Based Hydrogels

The selection and optimization of HA-based hydrogel formulations for bone regeneration applications are critical for achieving the desired mechanical and biological properties. The choice of cross-linking agent, cross-linking density, and HA concentration can have a significant impact on the properties of the resulting hydrogel.

**Figure 2:**
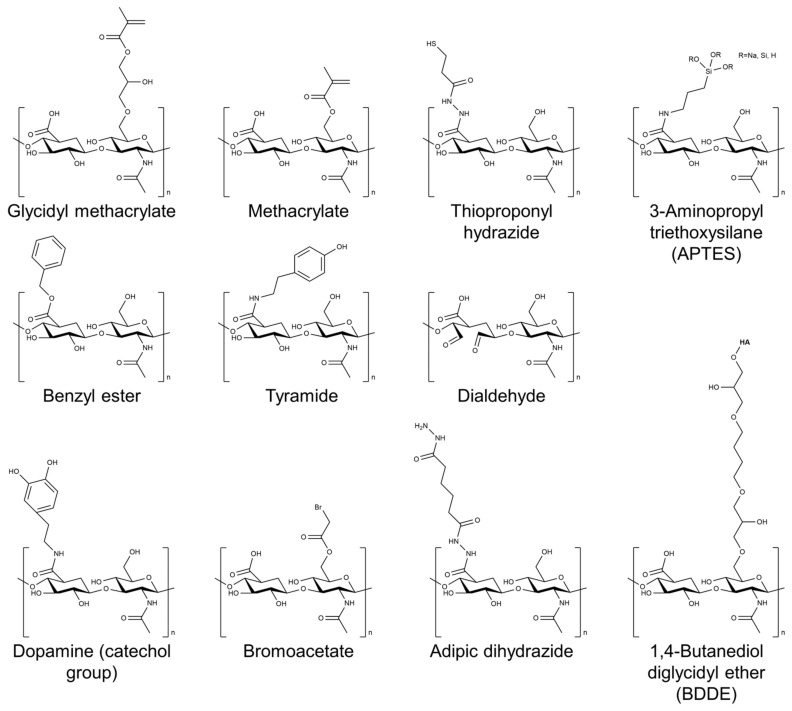
Chemical structure of HA modified with selected chemical cross-linking points for gelation of HA.

Several cross-linking agents and strategies have been used to cross-link HA-based hydrogels, including physical and chemical cross-linking agents [26,27,28]. Physical cross-linking includes methods such as thermal, pH-sensitive, and photomediated cross-linking, which rely on the reversible formation of physical cross-links between the HA chains. Chemical cross-linking agents such as glutaraldehyde, genipin, and carbodiimide form covalent bonds between the HA chains. The choice of cross-linking agent can have a significant impact on the properties of the resulting hydrogel. Physical cross-linking agents generally tend to result in hydrogels with lower mechanical strength but higher swelling capacity, whereas chemical cross-linking agents tend to result in hydrogels with higher mechanical strength but lower swelling capacity. Figure 2 shows the chemical structure of HA modified with selected chemical cross-linking points for gelation. Chemical modifications are mainly performed by targeting the carboxylic acid of the glucuronic acid residue or the C-6 hydroxyl group of the N-acetylglucosamine sugar of the HA backbone. In particular, approaches of chemical modification through addition, disulfide, enzyme, click reaction, and hydrazide are mainly used to form HA-based hydrogels without additional initiators [29].

In addition to the cross-linking agent, the concentration of HA in the hydrogel formulation can also affect the properties of the resulting hydrogel [30]. Higher concentrations of HA typically result in hydrogels with higher mechanical strength but lower swelling capacity. In addition, modifications to the cross-linking agent or the HA molecule itself can also be used to improve the properties of the hydrogel. The introduction of methacrylate or aldehyde groups onto the HA molecule has also been shown to enhance the mechanical properties of the resulting hydrogel [31,32,33].

To enhance the mechanical and biological properties of HA-based hydrogels for bone regeneration applications, several strategies have been investigated. One approach to improve the mechanical properties of HA-based hydrogels is to incorporate reinforcing agents into the hydrogel matrix. Reinforcing agents can provide additional strength and stiffness to the hydrogel, allowing it to withstand mechanical loading better. Examples of reinforcing agents that have been used in combination with HA-based hydrogels include various types of nanoparticles, such as carbon nanotubes, graphene oxide, and hydroxyapatite nanoparticles, as well as micro- and nanofibers made of biodegradable polymers such as poly(lactic-co-glycolic acid) (PLGA) and polycaprolactone (PCL) [34,35,36,37]. The incorporation of these reinforcing agents can significantly improve the mechanical properties of HA-based hydrogels, as demonstrated by studies showing increased compressive and tensile strength, modulus, and toughness [35]. In particular, the addition of bioceramics, such as hydroxyapatite or tricalcium phosphate, can improve the mechanical properties of the hydrogel and enhance its osteoconductive properties [38,39].

In addition to enhancing the mechanical properties of HA-based hydrogels, the incorporation of growth factors and other bioactive molecules can also improve their biological properties, such as their bioactivity and osteoinductivity. Growth factors such as BMPs, TGF-β, and platelet-derived growth factor (PDGF) have been shown to enhance bone regeneration when incorporated into HA-based hydrogels [39]. Similarly, other bioactive molecules such as ECM proteins, such as collagen and fibronectin, as well as small-molecule drugs such as dexamethasone and simvastatin, have been shown to improve the biological properties of HA-based hydrogels [16,40]. The incorporation of bone morphogenetic protein-2 (BMP-2) and stromal cell-derived factor-1α (SDF-1α) into an HA-based hydrogel was shown to promote bone formation and accelerate bone regeneration in vivo [41].

HA-based hydrogels can be formed into various shapes, such as particles, films, and porous scaffolds, using different fabrication techniques [38]. Advanced fabrication techniques, such as 3D printing, have been explored to create complex and functional HA-based scaffolds for bone regeneration [29,42].

## 3. Delivery of Bioactive Agents Using Hyaluronic-Acid-Based Hydrogels

In the field of bone tissue engineering, the development of biomaterials capable of delivering bioactive agents is of great interest. HA hydrogels possess several desirable properties, including biocompatibility, biodegradability, and the ability to retain a high water content, resembling the ECM of native tissues [29]. Moreover, HA possesses intrinsic bioactivity, promoting cell adhesion, migration, and proliferation [43]. The incorporation and delivery of bioactive agents within HA hydrogels can further enhance their therapeutic potential for bone regeneration [44].

Several strategies have been employed to achieve efficient delivery of bioactive agents within HA-based hydrogels [45]. These methods include physical entrapment, covalent immobilization, and affinity-based interactions. Each approach offers unique advantages and challenges in terms of controlling release kinetics, preserving bioactivity, and achieving spatiotemporal control over bioactive agent delivery [45,46,47].

Physical entrapment involves the incorporation of bioactive agents directly within the hydrogel matrix during gelation [42,48]. This method is relatively simple and versatile, allowing for the encapsulation of a wide range of bioactive agents, including growth factors, peptides, proteins, and nanoparticles. However, release kinetics can be difficult to control, potentially leading to burst release or insufficient delivery. Various factors, such as hydrogel cross-linking density, agent concentration, and hydrogel composition, can be optimized to modulate release kinetics and achieve sustained delivery [49,50,51].

Covalent immobilization involves chemically linking bioactive agents to the HA backbone through covalent bonds [42,52,53]. This approach provides controlled release kinetics and stability, ensuring the bioactive agent’s localized delivery and sustained bioactivity. Common methods for covalent immobilization include functionalizing the HA backbone with reactive groups, such as amino groups or thiol groups, and coupling them with the bioactive agent via cross-linking or conjugation chemistry. Covalent immobilization allows for precise control over release kinetics; however, the conjugation process must be carefully designed to preserve the bioactivity of the agent [54,55].

Affinity-based interactions rely on noncovalent binding interactions between the bioactive agent and HA molecules [56,57]. These interactions can be electrostatic, hydrophobic, or specific receptor–ligand interactions. Affinity-based delivery systems can provide reversible and stimuli-responsive release of bioactive agents. The use of electrostatic interactions can allow for controlled release in response to changes in pH or ionic strength. Affinity-based interactions offer versatility in terms of controlling release kinetics, but careful consideration must be given to the stability and specificity of the binding interactions [58].

The incorporation of bioactive agents into HA-based hydrogels has shown great promise in enhancing bone regeneration (Table 1). Growth factors, including BMPs, PDGFs, insulin-like growth factors (IGFs), and fibroblast growth factors (FGFs), stimulate cellular activities and promote osteogenesis. Chemical agents, such as small molecules and inorganic ions, provide additional osteogenic cues. Genetic molecules, including plasmid DNA and small interfering RNA (siRNA), offer the potential to manipulate cellular behavior and enhance bone regeneration.

### 3.1. Growth Factors

**Figure 3:**
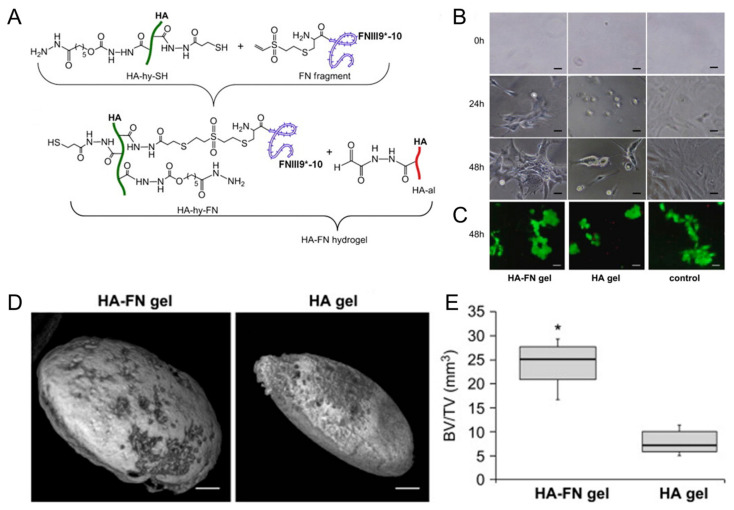
(**A**) Conjugation of fibronectin (FN III9*-10) to hyaluronic acid (HA) via Michael-type addition between vinyl sulfone groups of FN III9*-10 and thiol groups of HA (HA-hy-SH), then in situ cross-linking of the fibronectin-conjugated HA with aldehyde-modified HA to form HA–FN hydrogel via thioacetal formation. (**B**) Photographs of cell adhering and spreading on fibronectin-conjugated HA (HA–FN) and HA hydrogels. Scale bars indicate 50 μm. (**C**) Live/dead staining with calcein (live cells, green) and ethidium bromide (dead cells, red) on the hydrogels. Scale bars indicate 50 μm. (**D**) Ectopic bone formation 7 weeks post implantation of the hydrogels. Scale bars indicate 1 mm. (**E**) Quantification of bone volume normalized to the total tissue volume (BV/TV). * *p* < 0.01. Reproduced with permission from Kisiel et al. [63].

Growth factors play a crucial role in bone regeneration by modulating cellular behavior and tissue development. Various growth factors have been extensively studied for their osteogenic potential when incorporated into HA-based hydrogels. BMPs are members of the TGF-β superfamily and are known to induce osteoblastic differentiation and bone formation [40,74]. When incorporated into HA-based hydrogels, BMPs can promote MSC differentiation into osteoblasts, stimulate matrix deposition, and accelerate bone-healing processes [59,60,61,62]. Kisiel et al. reported a BMP-2 delivery system based on an integrin-specific ligand (fibronectin fragment, FN)-grafted HA hydrogel (Figure 3) [63]. This hydrogel enhanced the attachment and spreading of MSCs. A rat ectopic bone formation study resulted in enhanced bone formation and collagen fiber organization.

Recently, controlled delivery of BMP-2 using microfluidic-based pectin microparticles and a gelatin–elastin–HA hydrogel scaffold was reported for bone tissue engineering [64]. This hydrogel system achieved sustained delivery of BMP-2 and enhanced pro-osteogenic effect in vitro.

Growth and differentiation factor-5 (GDF-5), also known as bone morphogenetic protein-14 (BMP-14), has attracted significant attention for its potent osteogenic properties and its potential application in bone tissue engineering. Bae et al. developed a photomediated HA hydrogel containing GDF-5 for bone regeneration [65]. The evaluations of release profiles from the hydrogels showed a sustained release manner. In vitro cell-based assays resulted in enhanced cell proliferation and differentiation on the hydrogels. Bone regeneration using a rabbit calvarial defect model with a diameter of 8 mm significantly improved in the groups using GDF-5-incorporated HA-based hydrogels.

A click-cross-linking HA hydrogel was designed and developed from the click reaction of HA–tetrazine and HA–cyclooctene by a simple mixing process for bone tissue engineering [66]. BMP-2 mimetic peptides were incorporated into the hydrogel by chemically conjugating on the HA polymers. This strategy aimed for a more sustained release of BMP-2 mimetic peptides from the hydrogel compared with the physically loaded hydrogel groups, resulting in a prolonged retention time in vivo. In addition, in vivo study showed an enhanced formation of bone tissue.

### 3.2. Chemical Agents

Several chemical agents have been investigated for their role in bone regeneration when incorporated into HA-based hydrogels. Small molecules, such as simvastatin, have been incorporated into HA hydrogels to enhance bone regeneration. Simvastatin is an inhibitor of 3-hydroxy-3-methylglutaryl–coenzyme A reductase and is known as an efficient drug for osteoblastic differentiation of stem cells [75,76,77]. The Kwon group reported a photocuring hydrogel based on methacrylate-modified HA incorporating simvastatin for bone regeneration [67]. Simvastatin-incorporated HA hydrogel showed good biocompatibility, determined by MTT and live/dead assays in vitro, and a sustained release manner. A series of in vitro osteogenic activity and in vivo bone regeneration studies, including Alizarin Red S staining, PCR analysis for osteogenic-related genes, OCN and CPN, and X-ray radiography analysis, showed improved osteogenic activity and re-ossification.

### 3.3. Genetic Molecules

Genetic molecules, such as genes, plasmids, and siRNA, have attracted attention for their ability to manipulate cellular behavior and enhance tissue regeneration within HA-based hydrogels [40]. siRNA can be used to silence specific genes involved in inhibiting bone regeneration, such as inhibitors of the BMP pathway. Incorporating siRNA into HA hydrogels allows for localized and sustained gene silencing, leading to enhanced osteogenic differentiation and bone formation for the treatment of bone-related diseases [2]. Paidikondala et al. reported osteoinductive siRNA delivery using an aldehyde-modified HA hydrogel with acylhydrazide poly(vinyl alcohol) [68]. Hydrazone-cross-linked HA hydrogel incorporated with siRNAs against pleckstrin homology domain-containing family O member 1 (PLEKHO1) as a key causative of osteoporosis resulted in successful gene silencing and knockdown of protein expression with a low cytotoxicity in vitro [78].

### 3.4. Inorganic Ions

Inorganic ions, including calcium, phosphate, and strontium, have been incorporated into HA hydrogels to mimic the mineral composition of natural bone tissue [40,79]. These ions provide osteoconductive properties, promote osteoblastic differentiation, and enhance bone mineralization [79]. Lee’s group reported a biphasic calcium phosphate granule composite hydrogel based on HA and gelatin for bone regeneration [69,70,71]. The composite of the granule in an HA–gelatin hydrogel improved the mechanical strength and decreased swelling and degradation rates. In vitro and in vivo studies resulted in enhanced cell growth and proliferation of preosteoblast cells (MC3T3-E1) and good bone formation after implantation in a rabbit femur defect model. In addition, further incorporation of an autologous SVF in this composite hydrogel significantly enhanced the in vivo bone regeneration in a rat skull critical size defect model [70].

**Figure 4:**
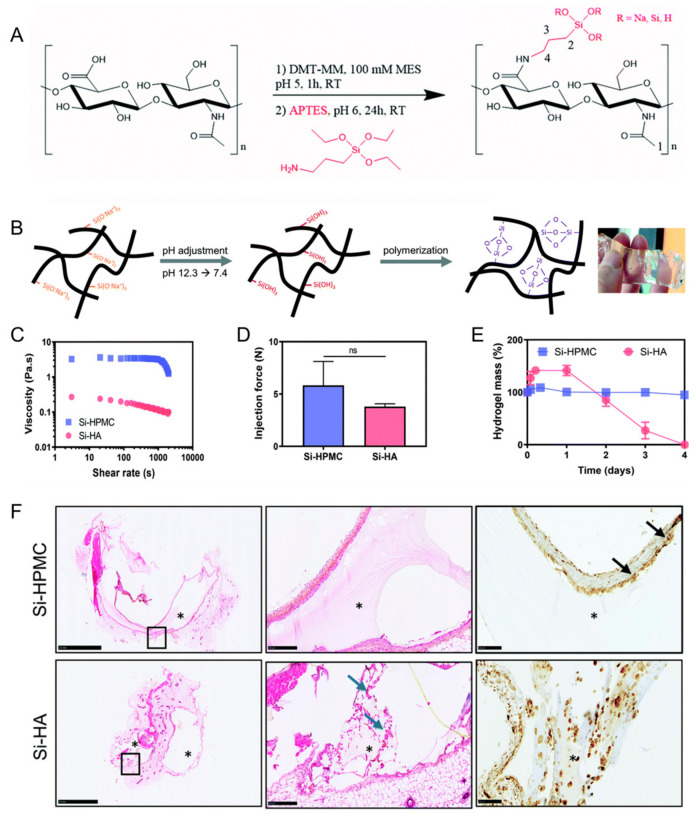
(**A**) Synthesis of silanized hyaluronic acid (Si–HA). (**B**) Illustration of Si–HA hydrogel formation processes. (**C**) Viscosity measurements of silanized hydroxypropylmethylcellulose (Si–HPMC) and Si–HA precursor solutions at 23 °C. (**D**) Injection (or extrusion) forces through an 18 G needle. (**E**) Degradation of the hydrogels in the presence of hyaluronidase. (**F**) Hematoxylin and eosin (H&E) staining and CD68 immunostaining 21 days post implantation. Scale bar: 100 μm. The second image is an enlargement of the black boxed portion of the first image. Blue arrows indicate new matrix secretion by fibroblasts. Black arrows indicate giant cells. *: Hydrogel. NS: not significant. Reproduced with permission from Flegeau et al. [72].

Flegeau et al. developed a biphasic calcium phosphate granule composite hydrogel based on silanized HA (Figure 4) [72]. This composite hydrogel had injectability and degradability. Enhanced bone healing of the degradable silanized HA composite hydrogel in vivo was determined in a rabbit knee defect model in comparison with a nondegradable silanized hydroxypropylmethylcellulose composite hydrogel.

Asensio et al. developed a biomimetic composite scaffold based on PEG dimethacrylate, PLGA, and methacrylated HA-hydrogel-incorporated β-tricalcium phosphate, strontium folate, and zinc folate with a hierarchical design [73]. This scaffold released bioactive inorganic compounds, strontium ions, and zinc ions in a sustained manner for 3 weeks within a biologically active range. In vitro studies revealed successful support of cell colonization and proliferation. The regeneration of osteochondral tissue was promoted by this scaffold in a rabbit condyle critical size defect model.

## 4. Combination of HA-Based Hydrogels with Other Biomaterials

The combination of HA-based hydrogels with other biomaterials has been explored as a means to enhance the properties of hydrogels for bone tissue engineering applications. Several biomaterials have been investigated for their potential to improve the mechanical properties, osteogenic potential, and biocompatibility of HA-based hydrogels. In this section, we discuss some of the biomaterials that have been investigated for combination with HA-based hydrogels (Table 2).

### 4.1. Collagen

Collagen is the most abundant protein in the ECM of bone tissue and has been extensively studied for its potential in bone tissue engineering applications [95,96]. Among 28 types of collagen, type I collagen is known to be most abundant in the ECM, especially in bones [97]. In addition, collagen is a feasible biomaterial that has excellent biocompatibility, degradability, low immunogenicity, and osteogenic induction properties [98]. The combination of HA-based hydrogels with collagen has been shown to improve the mechanical properties and osteogenic potential of hydrogels. Collagen provides an excellent matrix and can act as a structural component for 3D cell culture to improve the mechanical properties of hydrogels while also providing a favorable environment for cell attachment and proliferation [95,98,99]. The combination of HA-based hydrogels with collagen has been shown to enhance the expression of osteogenic markers in vitro and improve bone regeneration in vivo.

Gilarska et al. reported an injectable hydrogel based on collagen, chitosan, and lysine-functionalized HA for potential bone tissue engineering applications with multifunctional properties, such as a tunable physicochemical property, biocompatibility toward osteoblast-like MG-63 cells, antibacterial activity, and osteogenic activity [80].

**Figure 5:**
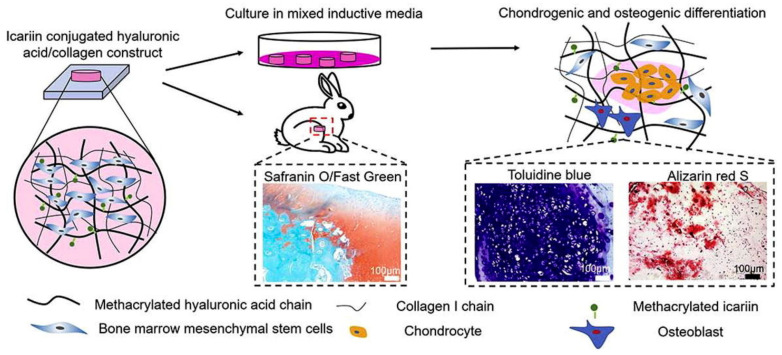
Icariin-conjugated HA hydrogel with collagen for osteochondral tissue regeneration. Reproduced with permission from Yang et al. [81].

Yang et al. developed icariin-conjugated HA hydrogel with collagen for osteochondral tissue regeneration (Figure 5) [81]. In vitro bioactivity tests showed that the HA/collagen hydrogel with icariin enhanced chondrogenesis and osteogenesis, resulting from better gene expression and calcium deposition. Furthermore, this hydrogel exhibited the reconstruction of osteochondral regeneration in a rabbit subchondral defect model.

### 4.2. Chitosan

Chitosan is a natural polymer derived from chitin, which is found in the exoskeleton of crustaceans [100]. Chitosan has been investigated for its potential in bone tissue engineering applications due to its superior biocompatibility, biodegradability, bioactivity, nontoxicity, antibacterial, and antifungal properties [101,102,103,104,105]. Chitosan can act as a structural component to improve the mechanical properties of hydrogels while also promoting cell proliferation and differentiation [80,106]. The combination of HA-based hydrogels with chitosan has been shown to improve bone regeneration in vivo.

**Figure 6:**
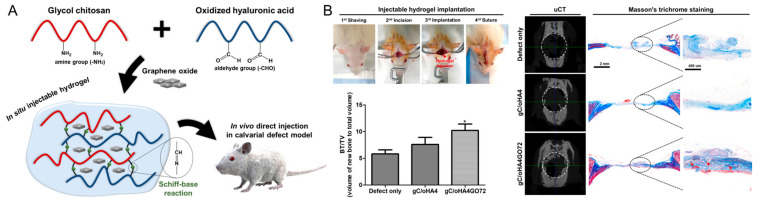
(**A**) Schematic illustration of the graphene-oxide-incorporated glycol chitosan and hyaluronic acid composite hydrogel for bone tissue engineering. (**B**) Implantation procedure of injectable hydrogels and in vivo bone-healing results, including new bone volume (bone volume/tissue volume, BV/TV), micro-CT reconstruction images, and Masson’s trichrome staining. * *p* < 0.05 as compared to defect only group. Reproduced with permission from Lee et al. [83].

A composite hydrogel of HA, glycol chitosan, and nanohydroxyapatite was developed for bone tissue engineering by Huang et al. [82]. The resulting hydrogels showed decreased porosity and swelling with an increase in HA content. In vitro cytocompatibility and cell adherence/spreading were evaluated in the hydrogel. Furthermore, Lee et al. developed a graphene-oxide-incorporated chitosan and HA composite hydrogel for bone regeneration (Figure 6) [83]. The composite of graphene oxide and glycol chitosan showed a robust mechanical property and stability. A series of in vivo evaluations exhibited good cytocompatibility and improved osteogenic activity, determined from ALP and Alizarin Red S stainings, immunostaining, and PCR assay. New bone formation in vivo was improved with the implantation of the resulting hydrogel in a rat calvarial defect model.

### 4.3. Silk Fibroin

Silk fibroin is a natural protein derived from silk fibers and has been investigated for its potential in bone tissue engineering applications due to its biocompatibility, biodegradability, and its unique mechanical properties [107]. Silk fibroin is also known to support the differentiation of MSCs along the osteogenic lineage; thus, it can be processed into hydrogels, particles, microspheres, and scaffolds for bone tissue engineering [107,108,109,110].

**Figure 7:**
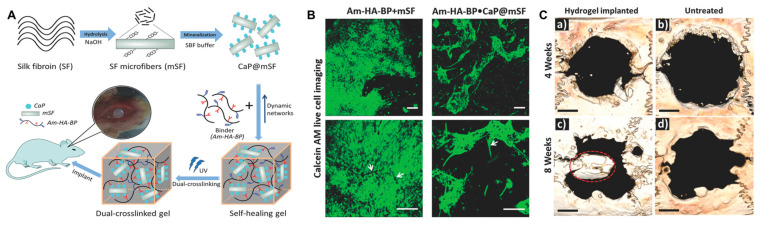
(**A**) Schematic illustration of a dual cross-linked hydrogel based on calcium phosphate, silk fibroin, and HA for bone tissue engineering. SF, silk fibroin; mSF, SF microfiber; CaP, calcium phosphate; CaP@mSF, CaP-coated mSF; Am–HA–BP, acrylamide and bisphosphonate-conjugated HA. (**B**) Live cell imaging post staining with calcein AM (green) of single cross-linked (Am–HA–BP+mSF) and dual-cross-linked (Am–HA–BP+CaP@mSF) hydrogels. Scale bars represent 200 μm. (**C**) New bone formation of the hydrogel determined using micro-CT in a rat cranial critical defect model. (**a**,**c**): hydrogel implantation. (**b**,**d**): blank (without hydrogel placement). Scale bars indicate 2 mm. Red circle indicates a newly formed bone flap. Reproduced with permission from Shi et al. [86].

Gokila et al. prepared tripolymeric scaffolds via a combination of nanochitosan/silk fibroin/HA ternary blends for bone regeneration and bone tissue formation [85]. In this study, the in vitro test cells favored the early adhesion, growth, and proliferation of preosteoblast cells. In addition, the scaffold facilitated osteogenic differentiation and calcium mineralization. Silk fibroin can act as a structural component to improve the mechanical properties of hydrogels while also promoting cell adhesion and proliferation. Shi et al. designed a dually cross-linked silk-fibroin-based hydrogel and demonstrated stem cell proliferation in vitro and bone regeneration in vivo without any growth factors (Figure 7) [86].

Recently, Yu et al. reported an HA/silk fibroin hydrogel containing curcumin-incorporating chitosan nanoparticles for osteosarcoma treatment and bone regeneration [87]. The resulting hydrogel was fabricated using photocuring and ethanol treatment from methacrylated HA. In addition, pH-dependent and sustained release of curcumin was evaluated in vitro. In particular, a bifunctional effect for osteosarcoma treatment and osteoblast proliferation at one concentration of curcumin (150 mg/L) was determined. This hydrogel resulted in approximately 45% viability against osteosarcoma MG-63 cells, indicating that this hydrogel exhibited anti-cancer effects. However, in vitro proliferation of preosteoblast MC3T3-E1 cells was improved with this hydrogel with a significant difference in comparison with control groups.

### 4.4. Gelatin

Gelatin is a denatured form of collagen, which is a major component of ECM, and is applied in bone tissue engineering due to its biocompatibility, degradability, low antigenicity, and physicochemical stability along with the arginine–glycine–asparagine (RGD) sequence that is essential for cell differentiation, adhesion, and proliferation [111,112]. Gelatin can also act as a structural component to improve the mechanical, physical, and chemical properties of hydrogels while also promoting cell growth, adhesion, and proliferation [113]. In particular, cross-linking of gelatin can alter its mechanical properties and degradation time by controlling the cross-link densities [114].

**Figure 8:**
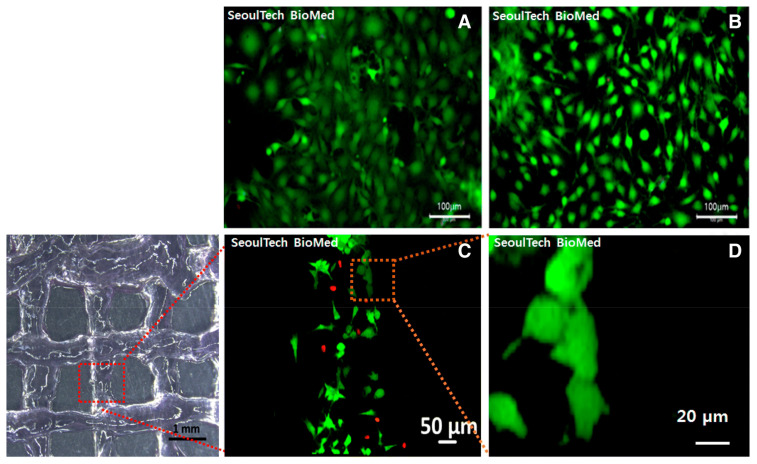
(**A**,**B**) Fluorescence images of live (green) and dead (red) assay in poly(hydroxyethyl acrylate)-conjugated HA and gelatin methacryloyl hydrogel after 3D bioprinting with bone cells. (**C**,**D**) Fluorescence images of live and dead assay 1 day post culture. Reproduced with permission from Noh et al. [88].

The combination of HA-based hydrogels with gelatin has been shown to improve the mechanical properties and osteogenic potential of hydrogels. Noh et al. developed an injectable hydrogel as a 3D-printable bioink using poly(hydroxyethyl acrylate)-conjugated HA and gelatin methacryloyl and performed an in vitro bone cell study (Figure 8) [88]. The results demonstrated that bone cells were well proliferated and spread with stable rheology properties, which provided constructs to form new ECM with the cells.

Furthermore, the combination of BMP-2-conjugated carbon dots embedded in a gelatin–elastin–HA hydrogel scaffold was developed by the Mohseni group and demonstrated enhanced biological properties and pro-osteogenic effect [64]. In addition, it had a positive effect on bone cell differentiation and promoted osteoblastic cell functions such as mineralization for bone tissue regeneration.

### 4.5. Synthetic Polymers

PCL is a biodegradable polyester that has been extensively investigated for its potential in bone tissue engineering applications due to its availability, low price, and chemical modification potential, and the degradation of PCL is relatively slow, which indicates a long half-life and makes it a good candidate for hard tissue engineering [115]. It also provides a good matrix for the regeneration of bone tissue [116]. PCL is more hydrophobic, less absorbable, and more flexible than poly(lactic acid) (PLA), which provides a stable matrix in bone regeneration. The combination of HA-based hydrogels with PCL scaffolds has been shown to act as a delivery carrier of bioactive agents, provide a biocompatible environment, and improve biological properties, including osteogenic response [89,116,117].

Rachmiel et al. developed a scaffold composed of PCL and HA and incorporated a short peptide for bone tissue engineering [89]. In vitro studies showed that preosteoblasts adhered to and proliferated well on the scaffold with demonstrated enhanced osteogenic differentiation and calcium mineralization. Thus, a PCL-based scaffold was suggested as a favorable biocompatible scaffold in bone tissue regeneration.

**Figure 9:**
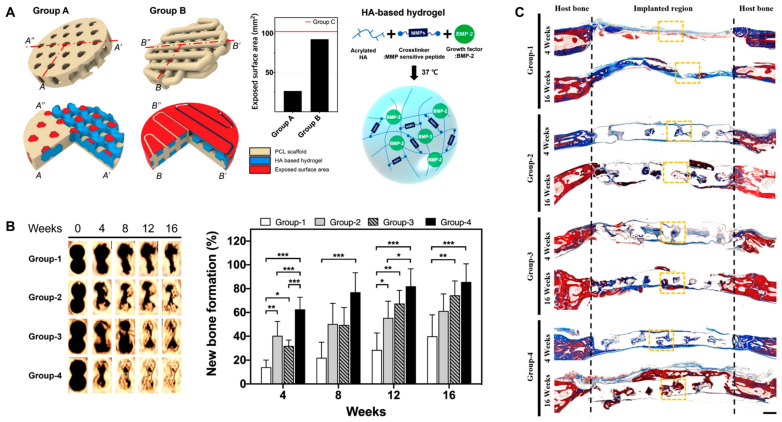
(**A**) Schematic illustration of PCL scaffold 3D structure and BMP-2-incorporated HA hydrogel. Group A, kagome-structure scaffold; Group B, grid-structure scaffold. The graph is the exposed surface area of the PCL scaffolds. (**B**) Three-dimensional live micro-CT images and new bone formation in a calvarial defect model. * *p* < 0.05, ** *p* < 0.01, *** *p* < 0.001. (**C**) Masson’s trichrome staining images at 4 and 16 weeks post implantation. The scale bar indicates 1 mm. The black dotted lines indicate the implanted area of the kagome-structure scaffold. The yellow dotted lines indicate the region of the magnified images. Group-1, nonimplanted defect; Group-2, kagome-structure PCL scaffold; Group-3, kagome-structure PCL scaffold with HA hydrogel; Group-4, kagome-structure PCL scaffold with HA hydrogel containing BMP-2 for (**B**,**C**). Reproduced with permission from Lee et al. [90].

Very recently, Lee et al. reported a kagome-structure PCL scaffold combined with a BMP-2-incorporated HA hydrogel by the 3D printing method for bone regeneration (Figure 9) [90]. The kagome structure enabled better retention of the hydrogel and sustained release of BMP-2. The combination of HA hydrogels enhanced new bone formation and further intensified with BMP-2 delivery.

Poly(ethylene glycol) (PEG) is a highly hydrophilic, biocompatible polymer that has been extensively investigated for its potential in tissue engineering applications due to its biocompatibility, biodegradability, and ability to form hydrogels [118]. Biodegradation of PEG hydrogel was dependent on PEG composition and showed tissue integration in rat models, which is important for the interaction of cells with ECM integrin receptors [119]. In addition, PEG has been used in combination with other polymers to regenerate several tissues, including bone [120]. The combination of HA-based hydrogels with PEG has been shown to improve in vitro and in vivo studies. Zhou et al. developed an injectable hyaluronan–methylcellulose hydrogel cross-linked with PEG, and in vitro tests demonstrated better cytocompatibility at a lower concentration of hydrogels [121]. Kwarta et al. designed an HA–PEG injectable hydrogel to restore disk thickness and hydration, and in vitro tests showed that the hydrogel stimulated disk generation with no cytotoxicity [122].

### 4.6. Nanoparticles

Nanoparticles are a type of particle with sizes ranging from 1 to 100 nanometers in diameter. Because of their small size, nanoparticles have unique properties that can be utilized for various applications, including drug delivery, imaging, and tissue engineering [123]. In the field of bone tissue engineering, nanoparticles have been investigated for their potential to improve the properties of HA-based hydrogels, including mechanical strength, osteogenic potential, and drug delivery capabilities.

Hydroxyapatite has chemical similarity to the bone matrix component and shows strong affinity to hard tissues; thus, it has been extensively investigated as a biomaterial for bone tissue engineering applications such as bone repair, bone augmentation, and fillers in bone or teeth [124]. Moreover, hydroxyapatite has been suggested as an ideal material for orthopedic and dental implants or components of implants due to its good biocompatibility and bone integration ability. Hydroxyapatite nanoparticles have been incorporated into HA-based hydrogels to improve their mechanical properties and osteogenic potential [125].

Arjama et al. reported that hydroxyapatite nanoparticles were incorporated into an HA-based hydrogel, and the resulting composite was shown to have better mechanical properties than the pure HA-based hydrogel and supported osteogenesis through a cell signaling cascade [91]. In this study, the hydrogel provided higher mechanical strength, particle uptake, and swelling efficiency, which enhanced cell attachment and biomineralization.

Dennis et al. reported that HA–hydroxyapatite colloidal gels combined with ECM displayed a superior storage modulus during rheological tests, yield stresses, and viscoelastic recovery compared with control groups [92]. In vitro assessment demonstrated that they promoted cell viability without cytotoxicity.

Jamnezhad et al. reported an HA hydrogel with alginate and titanium dioxide nanoparticles as a bone filler using a freeze-drying method for orthopedic application [93]. The addition of titanium dioxide nanoparticles improved the mechanical strength, wettability, and porosity of the hydrogel. The hydrogel was also usually nontoxic toward osteoblast cells except for at the highest incorporation of titanium dioxide nanoparticles.

**Figure 10:**
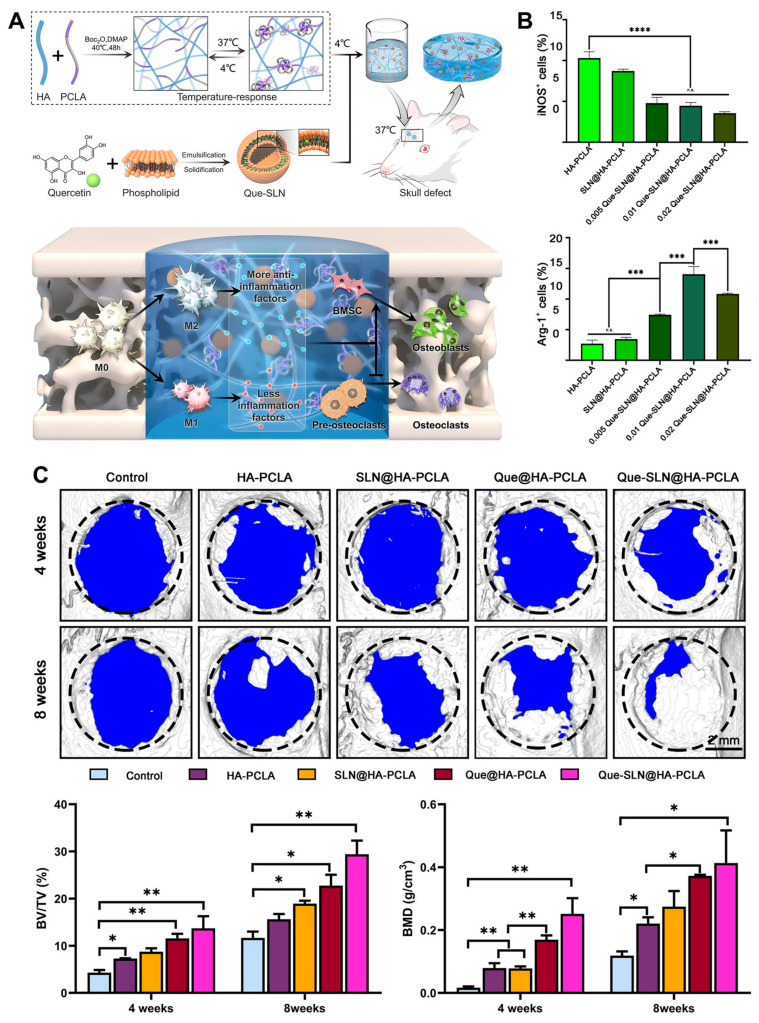
(**A**) Schematic illustration of bone repair by the HA hydrogel combined with a temperature-responsive synthetic polymer (PCLA) and quercetin-loaded solid lipid nanoparticle via macrophage immunoregulatory mechanism. (**B**) Immunoregulatory mechanism of hydrogel determined by quantitative analysis of immunofluorescence staining for M1 (iNOS^+^) and M2 (Arg-1^+^) macrophages. n.s.: not significant, *** *p* < 0.001, **** *p* < 0.0001. (**C**) Three-dimensional reconstructed images of skull defects using micro-CT analysis. Bone volume/tissue volume (BV/TV) and bone mineral density (BMD). * *p* < 0.05, ** *p* < 0.01. Reproduced with permission from Zhou et al. [94].

Very recently, an HA hydrogel combined with a temperature-responsive synthetic polymer and quercetin-loaded solid lipid nanoparticle was developed for bone tissue engineering (Figure 10) [94]. This HA-based hydrogel showed an anti-inflammatory effect resulting from a decrease in M1 polarization and an increase in M2 polarization. In addition, synergistic enhancement of osteoblastogenesis, anti-osteoclastogenesis, and angiogenesis was determined, and large bone defect reconstruction in a rat skull defect model was evaluated.

## 5. Three-Dimensional Printing of Hyaluronic-Acid-Based Hydrogels

Three-dimensional printing is a technique that uses a computer-aided design (CAD) model to create a three-dimensional object layer by layer [29,42]. Three-dimensional printing allows for precise control over scaffold design and pore structure, which is essential for creating a scaffold that can mimic the complex architecture of natural bone tissue. In addition, 3D printing enables the incorporation of multiple materials, including HA-based hydrogels, to create composite scaffolds with enhanced mechanical and biological properties. Another advantage of 3D printing is that it can create patient-specific scaffolds based on medical imaging data, which can improve the accuracy of implantation, increasing the success rate of bone regeneration. However, the main limitation of 3D printing is the relatively low resolution of the printed scaffolds, which can affect the mechanical properties and lead to structural defects [126]. In addition, the use of high temperatures and harsh chemicals during the printing process may affect the bioactivity of the HA-based hydrogel.

**Figure 11:**
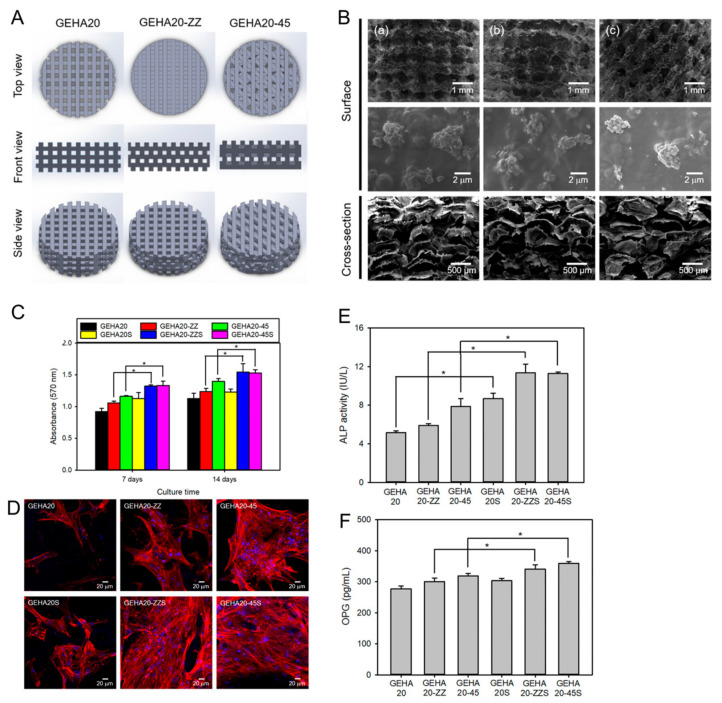
(**A**) Hyaluronic acid hydrogel 3D scaffold model for 3D printing. GEHA20, GEHA20-ZZ, and GEHA20-45 indicate the geometrical configurations of double-layer orthogonal, double-layer staggered orthogonal, and double-layer alternative scaffolds, respectively. (**B**) SEM images of various scaffolds (**a**) GEHA20S, (**b**) GEHA20-ZZS, and (**c**) GEHA20-45S) after mineralization with inorganic apatite crystals. (**C**) Cell proliferation of human mesenchymal stem cells on the scaffolds. (**D**) Confocal laser scanning microscopy images of human mesenchymal stem cells on the scaffolds after 7 days culture. (**E**) ALP activity after 14 days culture. (**F**) Osteoprotegerin (OPG) expression on the scaffolds after 14 days culture. * *p* ˂ 0.05. Reproduced with permission from Kim et al. [127].

Several studies have demonstrated the potential of advanced fabrication techniques to create HA-based scaffolds for bone regeneration. Kim et al. used 3D printing to fabricate an HA-based hybrid scaffold with a porous structure and interconnected pores for bone tissue engineering, as shown in Figure 11 [127]. The scaffold showed excellent biocompatibility and the ability to support the proliferation and osteogenic differentiation of human MSCs in vitro.

In another study, Patel et al. fabricated a composite hydrogel scaffold with methacrylated HA containing electrospun PCL nanofibers for bone tissue engineering [37]. The scaffold exhibited good mechanical properties and supported the growth and osteogenic differentiation of adipose-derived stem cells. In addition, a 3D-printed, HA-based scaffold was reported by Suo et al. [128]. They used a combination of HA, chitosan, and graphene oxide to create a porous scaffold with controlled porosity and mechanical properties. The scaffold was shown to support the adhesion and proliferation of mouse embryonic osteoblasts (MC3T3) and promoted their osteogenic differentiation in vitro.

## 6. Conclusions and Future Directions

This review showcased the extensive range of hydrogels derived from HA that have been developed in recent years, demonstrating the versatility in combining or synthesizing HA polymers and processing them with other nanomaterials and biomaterials with diverse properties. In addition, the incorporation of bioactive agents and the use of advanced fabrication techniques have allowed for the creation of complex and functional scaffolds that can mimic the native ECM of bone tissue and enhance bone regeneration. These hydrogels have proven to be highly valuable in various bone tissue engineering applications as well as drug delivery. Consequently, by developing multifunctional HA-based hydrogels, specific design criteria can be achieved. Moreover, HA-based hydrogels have shown the ability to influence cellular behavior, including stem cell differentiation, indicating their biological activity. One promising aspect is the potential application of HA-based hydrogels in translational research, primarily due to their processing capabilities, biocompatibility, and effectiveness. It is anticipated that the future will witness further expansion in this field through the development of novel materials with distinctive and intriguing properties.

However, there are still some challenges to be addressed before HA-based hydrogels can be translated into clinical applications. These challenges include regulatory and commercialization hurdles, as well as the need for further optimization of the mechanical properties and bioactivity of the hydrogels.

One strategy for addressing these challenges is to collaborate with regulatory agencies and industry partners to ensure that the safety and efficacy of HA-based hydrogels are thoroughly evaluated and properly marketed. In addition, continued research in the areas of biomaterials science, biochemistry, and tissue engineering will be crucial for the development of improved and more clinically relevant HA-based hydrogels.

Future research directions in this field include the development of more advanced and biocompatible fabrication techniques, such as bioprinting and electrospinning, as well as the use of new and innovative bioactive agents to enhance the performance of HA-based hydrogels. Furthermore, the use of these hydrogels in combination with other biomaterials and/or cell-based therapies may further enhance their regenerative potential and accelerate their translation into clinical applications.

Overall, HA-based hydrogels are a promising platform for bone tissue engineering and regenerative medicine. Continued research and collaboration are necessary to overcome the remaining challenges and unlock their full potential for clinical use.

## Figures and Tables

**Table 1:** Summary of bioactive agent delivery using hyaluronic-acid-based hydrogels.

Bioactive Agents	Materials	Key Findings	References
BMP-2	Aldehyde-modified hyaluronic acid, hydrazide-modified hyaluronic acid	Hydrazone-cross-linked hydrogel forming within 30 s. Nontoxic and controlled release of BMP-2. Improved in vivo bone regeneration.	[59]
BMP-2, vascular endothelial growth factor (VEGF)	Methacrylated hyaluronic acid with two different molecular weights, 220 and 110 kDa	Improved organized bone formation in the fastest and slowest degrading hydrogels. Further enhanced bone healing with co-delivery of VEGF.	[60]
BMP-2	Acrylated hyaluronic acid, tetrathiolated poly(ethylene glycol)	Improved cytocompatibility. Enhanced osteogenic factor expression and mature bone formation with vascular markers in vivo.	[61]
BMP-2	Thiol-modified hyaluronic acid, thiol-modified heparin, poly(ethylene glycol)-diacrylate	Sustained release of BMP-2 and further attenuation of BMP-2 release by the inclusion of heparin.Importance of tuning the initial burst of BMP-2.	[62]
BMP-2	Integrin-binding domain (fibronectin, FN III9*-10)-conjugated hyaluronic acid, aldehyde-modified hyaluronic acid	Improved stem cell attachment and spreading. Better bone formation and collagen fiber organization in vivo.	[63]
BMP-2 (carbon dot-conjugated BMP-2)	Pectin (microparticle), gelatin, elastin, hyaluronic acid	Sustained release of BMP-2. Cellular uptake of BMP-2 can be tracked by the carbon dot. Enhanced osteogenic effects.	[64]
Growth and differentiation factor-5 (GDF-5)	Methacrylated hyaluronic acid	Photocured hydrogel formation. Sustained release of GDF-5. Biocompatible and enhanced cell proliferation. Improved in vitro osteogenic activities and in vivo bone formation.	[65]
BMP-2 mimetic peptide	Tetrazine-conjugated hyaluronic acid, *trans*-cyclooctene-conjugated hyaluronic acid	Click-cross-linking hydrogel formation. Stable for a long period compared with unmodified hyaluronic hydrogel in vitro and in vivo. Biocompatible and improved in vitro osteogenic activities. Prolonged retention by chemical incorporation in the hydrogel.	[66]
Simvastatin	Methacrylated hyaluronic acid	Photocured hydrogel formation. Good biocompatibility. Sustained release of simvastatin. Improved in vitro and in vivo osteogenesis.	[67]
*Anti*-PLEKHO1 siRNA	Aldehyde-modified hyaluronic acid, hydrazide-modified poly(vinyl alcohol), Lipofectamine™ RNAiMAX	Approximately 90% of gene silencing and knockdown of protein expression with low cytotoxicity in vitro.	[68]
Biphasic calcium phosphate granules	Hyaluronic acid sodium salt, gelatin type A from porcine skin	Mechanical strength similar to cancellous bone substitute. Enhanced cell growth and proliferation. Better bone tissue regeneration.	[69]
Biphasic calcium phosphate granules, stromal vascular fraction (SVF)	Hyaluronic acid sodium salt, gelatin type A from porcine skin	Support of SVF heterogeneous cells on the surface of the hydrogel scaffold. Improved the bioactivity of the hydrogel scaffold with autologous SVF.	[70]
Biphasic calcium phosphate granules, platelet-rich plasma	Hyaluronic acid sodium salt, gelatin type A from porcine skin	Improved mechanical property with the calcium phosphate incorporation. Enhanced biocompatibility from platelet-rich plasma. No superior in vivo bone regeneration with the addition of platelet-rich plasma.	[71]
Biphasic calcium phosphate granules	Silanized hyaluronic acid	Easily injectable, fast hardening within 5 min. Biodegradable over 21 days. Improved in vivo bone regeneration.	[72]
β-tricalcium phosphate (β-TCP), strontium folate, zinc folic acid derivative	Poly(ethylene glycol) dimethacrylate, poly(d,l-lactide-*co*-glycolide), methacrylated hyaluronic acid	Sustained leaching of bioactive agents, strontium ions, zinc ions, and folic acids. Guided cell colonization and proliferation without negative effects in vitro. Improved in vivo neoformation of osteochondral tissue.	[73]

**Table 2:** Summary of hyaluronic-acid-based composite hydrogels with other biomaterials.

Hydrogel Formulation	Key Findings	References
Lysine-functionalized HA, collagen, chitosan	Altering physiological properties (swelling, wettability, enzymatic degradation, porosity, and rheology). Biocompatible and osteogenic potential. Antibacterial activity depending on the incorporation of chitosan.	[80]
Icariin-conjugated HA, collagen	Enhanced chondrogenesis and osteogenesis by icariin conjugation. Sustained release of icariin. Improved osteochondral regeneration in vivo.	[81]
HA (aldehyde-modified), glycol chitosan, nanohydroxyapatite	Porous structure associated with nanohydroxyapatites. Decreased porosity and swelling ratio with increased HA content. Enzymatic hydrolysis and degradation by lysozyme. Cytocompatible with MC3T3-E1 cells.	[82]
HA (aldehyde-modified by oxidation), glycol chitosan, graphene oxide	Robust mechanical properties and stability. In vitro cytocompatibility and enhanced osteogenesis. Enhanced in vivo rat calvarial defect repair.	[83]
HA, chitosan, graphene oxide, simvastatin	Enhanced osteogenic property by simvastatin delivery. Improved mechanical property by graphene oxide composite. Biocompatible and potentially osteoinductive.	[84]
HA, silk fibroin, chitosan/sodium tripolyphosphate nanoparticle	Good interconnected porous structure. Cytocompatible and enhanced osteogenic activity of osteoblasts. Potential antimicrobial effect.	[85]
Acrylamide and bisphosphonate-conjugated HA, calcium phosphate-coated silk fibroin microfiber	Enhanced mechanical property by dual cross-linking of coordination interaction and photopolymerization. Self-healing property. Biocompatible and accelerated bone regeneration.	[86]
Methacrylated HA, silk fibroin, curcumin-loaded chitosan/sodium tripolyphosphate nanoparticle	Pore size increase by a composite of silk fibroin and chitosan nanoparticles. pH-dependent and sustained release of curcumin. Bifunctional effect for osteosarcoma treatment and osteoblast proliferation.	[87]
HA-*g*-poly(hydroxyethyl acrylate), methacrylated gelatin	Stable rheology property and biocompatible. Potential 3D printing bioink. Potential bone cell delivery carrier.	[88]
Pectin (microparticle), gelatin, elastin, hyaluronic acid, BMP-2 (carbon dot-conjugated BMP-2)	Sustained release of BMP-2. Cellular uptake of BMP-2 can be tracked by the carbon dot. Enhanced osteogenic effects.	[64]
Hyaluronic acid sodium salt, gelatin type A from porcine skin, biphasic calcium phosphate granules	Mechanical strength similar to cancellous bone substitute. Enhanced cell growth and proliferation. Enhanced bone tissue regeneration.	[69]
Hyaluronic acid sodium salt, gelatin type A from porcine skin, biphasic calcium phosphate granules, stromal vascular fraction (SVF)	Support of SVF heterogeneous cells on the surface of the hydrogel scaffold. Improved bioactivity of the hydrogel scaffold with autologous SVF.	[70]
Hyaluronic acid sodium salt, gelatin type A from porcine skin, biphasic calcium phosphate granules, platelet-rich plasma	Improved mechanical property with calcium phosphate incorporation. Enhanced biocompatibility from platelet-rich plasma. No superior bone regeneration in vivo despite the addition of platelet-rich plasma.	[71]
HA, polycaprolactone (PCL), Fmoc-phenylalanine-arginine-glycine-aspartic acid	Fabricated by electrospinning technique with core/shell structure. Cell attachment support based on RGD motif composition. Biocompatible with preosteoblasts and enhanced osteogenic differentiation.	[89]
Acrylated HA, polycaprolactone, BMP-2	Three-dimensional printing-based scaffold fabrication. Enhanced osteoinductivity of the PCL scaffold by kagome structure, combination with the HA hydrogel, BMP-2 delivery in the HA hydrogel.	[90]
HA, silk fibroin, hydroxyapatite, hexapeptide glycine–alanine–glycine–alanine–glycine–X (X can be tyrosine, serine, or valine)	Enhanced mechanical property. Porous structure and enhanced biocompatibility. Enhanced osteogenesis. Antibacterial properties against *S. aureus* and *E. coli*.	[91]
HA, demineralized bone matrix, decellularized cartilage, hydroxyapatite nanoparticle	Improved rheological properties. Nontoxic and cytocompatible. Injectable through 18-gauge needles.	[92]
HA, alginate, titanium dioxide nanoparticles	Improved mechanical properties and wettability by the addition of titanium dioxide. Porous structure and usually nontoxic to osteoblasts.	[93]
HA, poly(ε-caprolactone-*co*-lactide)-*b*-poly(ethylene glycol)-*b*-poly(ε-caprolactone-*co*-lactide), quercetin solid lipid nanoparticle	Injectable bone immunomodulatory hydrogel. Sustained release of quercetin. Formation of an anti-inflammatory microenvironment by decreasing M1 polarization and increasing M2 polarization. Synergistic effect on angiogenesis and anti-osteoclastogenesis. Enhanced bone regeneration of large-scale bone defects.	[94]

## Data Availability

Not applicable.
